# Human Circulating miRNAs Real-time qRT-PCR-based Analysis: An Overview of Endogenous Reference Genes Used for Data Normalization

**DOI:** 10.3390/ijms20184353

**Published:** 2019-09-05

**Authors:** Simone Donati, Simone Ciuffi, Maria L. Brandi

**Affiliations:** 1 Department of Experimental and Clinical Biomedical Sciences “Mario Serio”, University of Study of Florence, Viale Pieraccini 6, 50139 Florence, Italy (S.D.) (S.C.); 2 Unit of Bone and Mineral Diseases, University Hospital of Florence, Largo Palagi 1, 50139 Florence, Italy

**Keywords:** circulating microRNAs, endogenous reference genes, non-invasive diagnostic biomarkers, real-time qRT-PCR

## Abstract

miRNAs are small non-coding RNAs of about 18–25 nucleotides that negatively regulate gene expression at the post-transcriptional level. It was reported that a deregulation of their expression patterns correlates to the onset and progression of various diseases. Recently, these molecules have been identified in a great plethora of biological fluids, and have also been proposed as potential diagnostic and prognostic biomarkers. Actually, real time quantitative polymerase chain reaction is the most widely used approach for circulating miRNAs (c-miRNAs) expression profiling. Nevertheless, the debate on the choice of the most suitable endogenous reference genes for c-miRNAs expression levels normalization is still open. In this regard, numerous research groups are focusing their efforts upon identifying specific, highly stable, endogenous c-mRNAs. The aim of this review is to provide an overview on the reference genes currently used in the study of various pathologies, offering to researchers the opportunity to select the appropriate molecules for c-miRNA levels normalization, when their choosing is based upon literature data.

## 1. Introduction

To date, progress has been made in a great variety of diseases for diagnosis and treatment. However, there is still a long way to go to perform early detection in order to improve diagnosis and, consequently, the outcome of the patients (prognosis). Therefore, several research groups are currently working to identify more sensitive, novel and easy-to-detect biomarkers which could be used in the diagnosis and prognosis of various diseases, including cancers. The ideal marker must use a non-invasive, cost-effective, disease-specific approach capable of providing a reliable early diagnosis of the disease before the onset of clinical symptoms.

Among these, many studies have focused on circulating microRNAs (c-miRNAs), which could become new biomarkers, combined with the conventional diagnostic methods [1].

In 1993, the first miRNA, *lin-4*, which affects development in the *Caenorhabditis elegans* (*C. Elegans*) targeting protein *lin-14* mRNA, was discovered by the Ambros and Ruvkun research groups [2,3].

In the next two decades, miRNAs were found in plants, animals, protists (eukaryotic organisms), and viruses (but not in bacteria) [4].

These miRNAs are a theoretically evolutionarily-conserved class of small non-coding RNAs, approximately 18–25 nucleotides, that negatively regulate gene expression at the post-transcriptional level [5,6]. They account for about 1% of the human genome [7].

About 52% of human miRNAs are located in intergenic regions, 40% within intronic regions, and the remaining 8% are exonic [7]. It has been estimated that miRNAs are able to regulate at least 20–30% of human transcripts [8].

Notoriously, miRNAs interact with the 3′ untranslated region (3′-UTR) of mRNAs target to induce their degradation and/or translational repression [9]. However, it has also been reported that miRNAs can interact with other regions, including 5′-UTR, and coding sequence [10]. It has also been reported that these molecules interact with promoter gene regions to increase or inhibit their transcription, although more studies are required to fully understand this interaction [11,12,13].

These miRNAs are able to target numerous genes by their seed sequence (nucleotides 2–8), as well as by base pairing in the central region (nucleotides 9–12) resulting in an amplification of their biological effects [14]. The miRNA/mRNA binding can occur either by incomplete complementary, leading to repression of translation of target, or with a complete complementary, leading to degradation of the target [15,16].

The biogenesis of miRNAs is classified into canonical and non-canonical pathways.

The canonical biogenesis pathway is the main pathway, and it starts with the transcription of a long primary miRNA (pri-miRNA) by RNA polymerase II, and is then processed into precursor miRNA (pre-miRNA) by a microprocessor complex, consisting of the RNA binding protein DiGeorge Syndrome Critical Region 8 (DGCR8) and Drosha, a ribonuclease III enzyme [17]. The resulting pre-miRNA presents two nucleotides 3′ overhang. Subsequently, pre-miRNAs are actively exported to the cytoplasm by an exportin 5/RanGTP complex, and then processed by the RNase III endonuclease Dicer, resulting in a small double strand RNA of about 18–25 nucleotides [17,18]. This duplex miRNA is unwound, and the strand with less thermodynamic stability at the 5′ ends, named the guide strand, is incorporated into the RNA-induced silencing complex (RISC) forming the miRNA-induced silencing complex (miRISC) [19,20]. The interaction between the miRISC and mRNA target occurs via miRNA response elements (MREs), and hence, mediates either mRNA degradation or translational inhibition according to complementarity to the mRNA target [4].

The non-canonical pathway makes use of a different processing way for non-coding RNAs (ncRNAs) maturation. Some miRNA-like products originate by the DROSHA-independent pathway, such as short hairpin RNAs (shRNAs) or miRtons. Other miRNA-like molecules, such as Ago-2-miRs, mature not by Dicer but by AGO2 protein. Finally, molecules such as endo-siRNA are produced by DROSHA-/Dicer-independent pathways [4]. However, despite these different biogenesis pathways, they all act as post-transcriptional regulators of gene expression.

Currently, there are 2812 human miRNA sequences listed in the miRNA registry (Available online: http://www.mirbase.org).

It is known that miRNAs are involved in many biological processes, including cell proliferation, differentiation, survival and others [21]. Therefore a deregulation of their expression patterns could result in the onset and progression of various diseases. Indeed, numerous studies have demonstrated that normal and pathological tissues can be distinguished based on the specific miRNAs’ expression profile. However, their clinical application as diagnostic and/or prognostic tools has never taken off, due to their invasive nature.

This substantially changed in 2008, when for the first time four independent studies [22,23,24,25] demonstrated the presence of stable form cell-free miRNAs in blood, and that their serum/plasma levels could be correlated to both specific physiological and pathological conditions [26], and therefore, potentially usable as non-invasive biomarkers [21].

Subsequent studies have demonstrated that miRNAs can be released into various other extracellular biofluids, such as cerebrospinal fluid [27], saliva [28], breast milk [29], urine, tears, bronchial lavage, seminal fluid [30] and ovarian follicular fluid [31].

These circulating small molecules have proven to be highly stable, resisting deleterious conditions such as boiling, multiple freeze-thaw cycles and high or low pH (alkali or acidic) [24,25]. Moreover, c-miRNAs are resistant to the high endogenous RNase activity present in the blood, suggesting that these small molecules adopt some protective mechanisms to bypass these adverse conditions [32].

Nevertheless, the mechanisms underlying their remarkable stability in blood and other biofluids remain unresolved. One theory suggests that c-miRNAs might be associated with proteins, such as the Ago family [33], high-density lipoproteins (HDLs) and nucleophosmin 1 (NPM1) [34,35,36]. Another assumption underlying the stability of c-miRNAs is that these molecules could be protected by inclusion into membrane vesicles, i.e., exosomes, microvesicles, and apoptotic bodies [26,28]. Particularly, exosomes have a diameter of 50–90 nm, and they are released into the extracellular environment as a result of the fusion of multivesicular bodies (MVBs) with the plasma membrane. Several studies report that reticulocytes [37], dendritic cells [38], B cells [39], T cells [40], mast cells [41], epithelial cells [42] and tumor cells [43] are able to release these vesicles of endocytic origin.

In the last decade, many studies have been performed demonstrating the potential role of c-miRNAs as biomarkers in several diseases [23,24,25,44,45]. However, to date their biological role remains unknown. Nevertheless, it has been proposed that these molecules are involved in intercellular communication mechanisms: Vesicle-associated miRNAs may enter cells by endocytosis, phagocytosis, or direct fusion with the plasma membranes, while proteins-associated miRNAs may be taken by specific receptors on the target cell surface [34,46].

To perform a correct biofluid miRNA analysis, it is first necessary to evaluate the sample source, and then the ideal methods of collection, preservation and processing. Finally, the methodology used for analysis expression and the strategy adopted for data normalization is also very important [47].

Different methodologies have been applied to qualitatively and quantitatively establish the signature miRNAs in body fluids, such as real-time qRT-PCR, microarray platform, northern blotting, ultra-high throughput miRNA sequencing, in situ hybridization with locked nucleic acid probes, hybridization in solution with tagged probes and droplet digital PCR [48]. The first approach allows the detection of a large specific set of miRNAs simultaneously. Nevertheless, their quantification appears rather complicated; in fact, the aid of both computational biologists and bioinformatics able to use software such as geNorm [49], NormFinder [50], BestKeeper [51], and the comparative delta Ct method [52] is required to evaluate the gene expression stability, a necessary condition to be used as reference genes (RGs). Furthermore, this technology requires a great input of starting RNA [53,54]. The NGS platform allows the detection of a large set of miRNAs, but, unlike microarray methodology, it also allows the discovery of novel miRNAs when miRNome analysis is performed [55]. On the other hand, data analysis requires the support of both computational biologists and bioinformatics, and moreover, this technology still has limitations in the detection of low abundance miRNAs [56]. To date, given the difficulty in data interpretation of both the above-mentioned methods, a data validation step is still required using the real-time qRT-PCR (from here on named qPCR) procedure [57,58]. This method, commonly used for c-miRNA expression studies, is more sensitive and specific than previous approaches. The disadvantage of this technology, compared to the previous platforms, is the limited set of miRNAs that can be tested in each experiment [26,59].

When performing gene expression studies, variation in the amount of starting material, sample collection, RNA extraction, reverse transcription and amplification efficiency, may introduce bias, and consequently, could lead to misleading conclusions and impaired comparison between studies [60]. Given these concerns, the selection of reliable RGs is necessary and essential to normalize the expression data.

However, to date, also due to the peculiarities of these samples, there is no consensus for the best RGs to be adopted to normalize qPCR data. Initially, external synthetic miRNA molecules, named spike-ins, were widely used for this purpose, but recently, specialists have suggested the identification and use of specific endogenous miRNAs as RGs.

In this regard, several studies have evaluated and suggested the best RGs for biofluid miRNA level normalization in a variety of pathologies (see Table 1). In the next section, this review will be focused on the RGs adopted to date.

## 2. Best Identified miRNAs as Potential RGs

### 2.1. miR-16

miR-16 is the miRNA most frequently used as an RG, according to the literature. Indeed, this miRNA results in being relatively stable in various studies, and consequently, is recommended as a suitable RG for circulating miRNA investigations. Zhao et al. [61] compared whether c-miRNA were differentially expressed between the plasma samples of breast cancer patients, being 10 Caucasian American (CA) and 10 African American (AA) and 20 healthy controls (HC), using the microarray approach followed by qPCR data validation. The choice to use miR-16 as an endogenous control for data normalization was made based upon data available in the literature. The results show that two miRNAs, miR-181 and miR-1304, overlap between CA and AA, and their plasma levels were differentially expressed when compared with HC.

Heneghan et al. [62] investigated the potential role of whole blood miRNAs as breast cancer diagnostic biomarkers. 83 breast cancer patients were compared, versus 44 HC. The qPCR was carried out to quantify seven miRNAs, chosen for their significant relevance in breast cancer. miR-16 has been adopted as an RG because its blood levels were stable across all samples, while miR-195 and let-7a were up-regulated in breast cancer patients compared to HC, and therefore potentially usable as biomarkers for this pathology.

Roth et al. [63] evaluated the clinical utility of c-miRNAs as non-invasive biomarkers for the revelation and staging of breast cancer. Specifically, they examined the serum levels of four miR-10b, miR-34a, miR-141 and miR-155, found deregulated in this pathology. c-miRNAs expression was evaluated by qPCR and data normalization was performed by the ∆Ct method by using miR-16 as an endogenous RG, according to the manufacturer’s recommendations (Applied Biosystems, Thermo Fischer, Waltham, MA, USA). They concluded that the c-miRNAs investigated were up-regulated in the blood of breast cancer patients and associated with tumor progression.

Gharbi et al. [64] analyzed the expression levels of miR-16 together with SNORD38B, SNORD49A, U6, 5S rRNA, miR-423-3p, miR-191 and miR-193 in the sera of 28 Sulfur Mustard-exposed Veterans (SMV) and 15 HC, in order to be adopted as RGs. These miRNAs were chosen based on the literature, with the exception of SNORD38B and SNORD49A, which were recommended by Exiqon. After a first evaluation in qPCR, they identified the miR-423-3p as the most stable endogenous RG, then miR-103 and miR-16 ranked after.

The aim of the study by Wang and colleagues [65] was to establish the most stable RGs for the analysis of serum miRNAs in bladder cancer. In the first phase, they used MiSeq sequencing to select the candidate RGs in three pooling samples, including 10 patients with non-muscle invasive bladder cancer (NMIBC), 10 patients with muscle-invasive bladder cancer (MIBC) and 10 HC. According to specific inclusion criteria, (1. miRNA was expressed in all samples of the study population; 2. the mean Ct value < 35; and 3. no differentiality was expressed between the three groups), 10 miRNAs (miR-193a-5p, miR-191-5p, miR-16-5p, miR-10a-5p, miR-345-5p, miR-143-3p, miR-140-3p, miR-502-3p, let-7d-3p and miR-141-3p) were selected for the subsequent qPCR evaluation phase. Furthermore, U6, identified as a suitable RG in the previous study, has also been added. Moreover, the serum of 30 patients with MIBC, 30 patients with NMIBC and 35 HC were used. Five molecules (miR-193a-5p, miR-16-5p, U6, miR-191-5p and let-7d-3p) were selected for the next stability evaluation phase. Then miR-193a-5p and miR-16-5 were revealed as the most stable combination for the analysis of serum miRNAs in bladder cancer. These results were confirmed in an independent cohort composed of 46 bladder cancer patients and 46 HC.

Wang et al. [66] evaluated the expression of miRNAs in the serum of 22 patients with osteogenesis imperfecta to elucidate their potential as a biomarker for this pathology. They used qPCR to identify the best internal RGs between miR-16, miR-638, snRNAU6, 18S rRNA, Let-7a and miR-92a. The last two were selected because they were stably expressed in previous experiments, while the others were common internal RGs. Although qPCR data had shown that all of these molecules were stable, stability software analysis revealed miR-16, snRNAU6, miR-92a and Let-7a as RGs, to normalize serum miRNAs levels in this pathology.

Song et al. [67] attempted to identify a reliable RG for the analysis of serum miRNAs in gastric cancer. They evaluated six miRNAs (let-7a, miR-16, miR-93, miR-103, miR-192 and miR-451) and one small nuclear RNA (RNU6B) according to literature searches and suggestions from Exiqon. Their expression levels were analyzed by qPCR in the serum samples of 20 gastric cancer patients and 20 HC. The use of miR-16 and miR-93, alone or combined, was found to be the most stable reference system.

In their studies, Wang et al. [68] evaluated the suitability of five candidate miRNAs (miR-16, miR-15b-5p, miR-24-3p, miR-19b-3p and let-7i-5p), chosen according to studies of literature, as potential RGs for c-miRNA normalization in serum samples from 35 patients with cardiovascular disease and 17 HC. In addition, they included two other commonly-used small, nuclear RNAs: SNOU6 and 5S. Applying stability analysis by using specific softwares to the qPCR data established that let-7i and miR-16 were the most recommended candidate RGs in hypertension and heart failure diseases, followed by miR-15b, miR-19b and miR-24-3p. Contrarily, SNOU6 and 5S were not reliable RGs.

### 2.2. miR-93

miR-93 has also been identified as a suitable RG to normalize c-miRNA levels in many pathologies, including gastric cancer, major depression, vulvar intraepithelial neoplasia lesions and vulvar carcinoma, tuberculosis and colorectal cancer.

Zalewski et al. [69] selected six appropriate RGs (miR-93-5p, miR-425-5p, miR-191-5p, miR-423-5p, miR-103a-3p and miR-16-5p) to normalize c-miRNA expression by using a qPCR assay. These miRNAs were chosen based on literature data which showed stable levels in a wide variety of biological tissues and body fluids. Plasma miRNA levels of 17 patients with vulvar intraepithelial neoplasia lesions were compared versus 27 vulvar squamous cell carcinoma patients. The plasma expression levels of six miRNAs were quantified by using qPCR. miR-93-5p and miR-425-5p resulted in being the best internal controls in plasma of vulvar intraepithelial neoplasia lesions and vulvar squamous cell carcinoma patients.

The purpose of the study of Liu et al. [70] was to identify reliable RGs for the analysis of miRNA expression in the plasma of major depressive disorder (MDD) patients by using qPCR. They performed a pre-selective miRNA expression profile by using a microarray platform. Based on four inclusive criteria, (1. miRNAs have to be present in all samples analyzed; 2. candidate miRNAs must be annotated in miRbase; 3. the fold change between the MDD patients and HC is to be lower than 1.1; and 4. no significant difference existed between the groups), they selected miR-93-5p as well as miR-320d, miR-101-3p, miR-106a-5p and miR-423-5p as candidate RGs. Moreover, a commonly-used RG, U6 small nuclear RNA (U6-snRNA), was also evaluated. These selected RGs were then validated by using a qPCR assay. The results led the authors to conclude that the combined use of miR-93-5p and miR-101-3p was the most robust tool for the c-miRNAs normalization of MDD patients.

Barry et al. [71] investigated the most suitable RGs for normalizing plasma miRNA levels by qPCR assays. Two cohorts of tuberculosis (TB) patients: Australian cohort, 12 patients with TB and 12 patients with latent TB infection (LTBI); Chinese cohort, 12 patients with TB and 12 patients with LTBI. The authors analyzed the plasma expression levels of twelve miRNAs (let-7, miR-16, miR-22, miR-26, miR-93, miR-103, miR-191, miR-192, miR-221, miR-423, miR-425 and miR-451) and RNU6B based on previous literature reports, by using qPCR.

The results have revealed miR-93 and miR-425 as the most stable RGs in Chinese patients, while miR-93, miR-221 and let-7a were the most stable normalization factors in the Australian patients. Furthermore, miR-93 and miR-22 have been shown to be the most stable RGs in the combined cohorts. In conclusion, miR-93 was the most stable reference miRNA, since its levels remained stable across diverse ethnic and geographic positions.

Niu et al. [72] analyzed the expression levels of 485 miRNAs by the qPCR approach in the pooled serum of 66 colorectal cancer patients and 86 HC, as a pre-screening phase. Seven of 485 miRNAs (miR-320d, mir-25-3p, miR-92b-3p, miR-106b-5p, miR-10b-5p, miR-107 and miR-574-5p) met the established criteria by the authors: 1. The Ct values of all miRNA were < 32; 2. the fold change between the colorectal cancer patients and healthy populations was lower than 1.1; 3. no significant miRNA expression difference between the diseased and healthy groups (*p*  >  0.05). These miRNAs were selected for the subsequent validation phase in an independent population (30 colorectal cancer patients and 30 HC randomly chosen from the initial population). Moreover, three miRNAs, miR-93-5p, miR-101-3p and miR-16-5p, were chosen as suitable RGs for qPCR analysis according to literature. The qPCR carried out for data validation showed miR-93-5p, miR-25-3p and miR-106b-5p as suitable RGs to normalize serum miRNAs levels of colorectal cancer patients.

### 2.3. Combination of miR-221, miR-26a

Li et al. [73] studied reliable RGs to analyze serum exosomal miRNAs expression levels by using qPCR in patients of primary hepatocellular carcinoma (HCC) with hepatitis B infection following surgical treatment. The authors investigated the expression stability of eight miRNAs (miR-16, miR-103, miR-191, let-7a, miR-26a, miR-221, miR-181a and miR-451) and two small RNAs (5S and U6), according to previous reports of their reliability as RGs for qPCR assay of hepatopathy patients. The stability algorithms applied to qPCR data, recommended miR-221, let-7a and miR-26a as the most stable set of RGs for c-miRNAs evaluation in liver carcinoma resection studies.

The aim of the study of Zhu et al. [74] was to identify suitable RGs for an analysis of serum miRNAs in patients with Hepatitis B Virus-Infected Patients. A TaqMan low density array pre-screening was performed to evaluate the miRNA signature in the disease. For this study, serum samples from 52 patients with chronic Hepatitis B Virus and 57 HC were tested. The pre-screening test identified ten potential miRNAs (miR-495, miR-26a, miR-221, miR-671-3p, miR-335, miR-27a*, miR-630, miR-22*, miR-181a-2* and miR-30e), which were selected to be validated by qPCR. In addition to these candidate RGs, U6, RNU6B and miR-16 were investigated based upon the literature. The stability ranking revealed the combination of miR-26a, miR-221 and miR-22* as the most appropriate internal RGs for this experimental set.

### 2.4. miR-191

miR-191, alone or in combination with other molecules, has been used as RG in some diseases, i.e., HCC, colorectal adenocarcinoma, and breast cancer. Li et al. [75] analyzed the serum exosomal levels of miR-26a, miR-21, miR-22*, miR-181a, miR-181c, miR-16, miR-103, miR-191, let-7a, 5SrRNA and U6snRNA by using qPCR. These candidate miRNAs were selected according to the literature. The statistical analysis revealed that the combination of miR-221, miR-191, let-7a, miR-181a and miR-26a is the best normalization set for analyzing serum exosomal miRNAs in patients with Hepatitis B or HCC by qPCR.

Zheng et al. [76] studied the suitable RGs in order to have a proper normalization of serum miRNA levels in colorectal adenocarcinoma patients. First, a pre-screening assessment on three pooled serum samples (30 patients with colorectal adenocarcinoma, 25 patients with colorectal adenoma, and 30 HC) was performed by using the Miseq system. According to the following criteria, 1. miRNA having at least 50 copies in the three pooled serum samples, 2. showed no differential expression among three groups, the authors selected a list of 13 miRNAs (miR-93-5p, miR-103b, miR-484, miR-16-5p, miR-3615, miR-18a-3p, miR-197-3p, miR-191-5p, miR-151a-3p, miR-26a-5p, miR-4446-3p, miR-221-3p and miR-3184-3p) for further investigation by qPCR. This was carried out in an independent cohort, including 45 colorectal adenocarcinoma patients, 40 colorectal adenoma patients, and 40 HC. The results evidenced miR-191-5p as the most stable RG and revealed miR-191-5p and U6 as the most steady pair of RGs to normalize serum miRNAs by using qPCR assay in both colorectal adenocarcinoma and adenoma.

In this study, Hu et al. [77] aimed to identify the best serum miRNA for the non-invasive diagnosis of breast cancer. First, they performed a pre-screening phase to search for reliable RGs to normalize c-miRNA expression by using Solexa sequencing and TaqMan low density array on 10 pooled serum samples, including two lung cancer pooling samples, two breast cancer pooling samples, one cervical cancer pooling sample, two gastric cancer pooling samples, one HCC pooling sample and two HC samples. The miRNAs that resulted to be most stable were selected for the validation phase in qPCR performed in an independent cohort (five esophageal cancer, five colon cancer, five rectal cancer, five pancreatic cancer, five oral cancer, five gastric cancer, five lung cancer, five breast cancer, five HCC and five HC). This two-step procedure allowed them to identify miR-191 and miR-484 as the best RGs to normalize serum miRNA levels by qPCR assay in breast cancer patients. These two molecules, together with cel-miR-39, were used to identify four specific c-miRNAs (miR-16, miR-25, miR-222 and miR-324-3p) as potential non-invasive prediction biomarkers for breast cancer development.

### 2.5. miR-320a

Allen-Rhoades et al. [78] aimed to identify specific non-invasive biomarkers for detection, therapeutic monitoring, and prognosis in osteosarcoma. However, this was very difficult due to the rarity of this pathology. To overcome this problem, they used engineered mouse models of osteosarcoma, trying to evaluate a c-miRNA signature for this pathology in humans. By utilizing a qPCR-based platform to analyze the plasma levels of 752 miRNAs in animal models, they identified four miRNAs (miR-205-5p, miR-214, miR-335-5p and miR-574-3p) as potential disease biomarkers. In the following human plasma data validation phase, by using the previous procedure, they first identified two RGs, miR-320a and miR-15a, and subsequently, by using these molecules for data normalization, observed that these four miRNAs were similarly dysregulated also in human models.

Schlosser et al. [79] tried to identify RGs in the plasma of pulmonary hypertension patients (PAH). Initially, a pre-screening of 1,066 miRNAs, using the qPCR method, was performed in the plasma of four PAH patients and three HC to establish a set of candidate RGs. The candidate reference miRNAs criteria were: 1. Be measurable in all samples, 2. show steady levels across plasma samples, and 3. have no known association with the disease under investigation. The stability ranking revealed miR-320a and miR-142-3p as the best pair of RGs. The suitability of the aforementioned candidates was validated in a second independent population composed of 13 HC and 14 PAH patients, confirming their reliability for normalizing qPCR data in this experimental set. 

Moreover, they evaluated the broad utility of these molecules by testing them in the plasma of septic shock patients. They measured a total of 372 predefined miRNAs. The combination of miR-10a and miR-320a, as well as miR-142-3p and miR-320a, were identified as the most stable RG pairs to normalize qPCR data in this pathology, suggesting that the combination of miR-142-3p and miR-320a for normalizing qPCR data may be valid in more than one disease context.

### 2.6. Other Genes Adopted as Reference

There are other miRNAs identified as potential RGs in literature, even if described in a single paper. The objective of Chen and colleagues [80] was to identify a universal novel biomarker for the diagnosis and prognosis of osteoporosis. Furthermore, they also sought to identify a universal RG to normalize serum miRNA levels in this pathology. For these purposes, they evaluated serum miRNA levels in various osteopenic and osteoporotic animal models, including rat, monkey and human. They first performed a microarray pre-screening test on pooling serum samples. Subsequently, a data validation step by real time qPCR, followed by RG stability evaluation, and osteoporosis biomarkers diagnostic value assessment by ROC curve analysis, was carried out. Overall, they identified miR-25-3p as a potential RG, miR-30b-5p as a potential biomarker able to distinguish normal from low BMD patients, and miR-103-3p, miR-142-3p and miR-328-3p as osteopenic/osteoporosis biomarkers.

An interesting study aimed to evaluate a stable RG for different types of cancers was carried out by Hu et al. [81] They first performed a microarray preliminary study to select the candidate RGs from the plasma of 171 cancer patients (57 HCC, 41 colorectal cancer and 73 lung cancer patients) and 80 HC. Six plasma miRNAs were indicated as the most stable miRNAs (miR-1228, miR-1225-3p, miR-30d, miR-939, miR-188-5p and miR-134). In addition, two commonly-used RGs from literature (miR-16 and miR-223) were selected. These eight miRNAs were subjected to a validation step in the plasma of an independent cohort of 92 cancer patients (31 HCC, 31 colorectal cancer and 30 lung cancer patients) and 92 control subjects (30 HC, 31 CHB patients and 31 cirrhosis patients). miR-1228 was suggested as the more stable RG. To ensure its utility as the RG for the quantification of c-miRNAs in cancer patients, its expression was further validated in 109 additional patients with other cancer types (23 esophageal cancer, 21 gastric cancer, 24 renal cancer, 20 prostate cancer (CaP) and 21 breast cancer patients). In conclusion, they suggested miR-1228 as a potential endogenous RG for normalizing plasma miRNA expression levels by qPCR in various types of cancers (HCC, colorectal cancer, lung cancer, esophageal cancer, gastric cancer, renal cancer, CaP and breast cancer patients).

The purpose of the study by Zhang et al. [82] was to discover a suitable RG to normalize qPCR data derived from plasma of stable coronary artery disease (CAD) patients. Four candidate miRNAs (hsa-miR-6090, hsa-miR-4516, hsa-miR-6089 and hsa-miR-3960) from the microarray analysis performed in plasma samples (8 CAD patients and 8 HC) were selected according to two criteria: 1. Expressed in all samples, and 2. highly expressed. Moreover, six commonly-used controls (RNU6, miR-16-5p, let-7d-5p, miR-484, miR-191-5p and miR-423) from the literature were also selected for a subsequent validation step by qPCR in 21 stable CAD patients and 21 HC. miR-6090 and miR-4516 were identified as candidate RGs from the stability analysis algorithms applied to qPCR data. These miRNAs were further validated in a larger cohort composed of 90 CAD patients and 90 HC. In conclusion, they recommended miR-6090 and miR-4516 as RGs to normalize plasma miRNAs levels in CAD patients.

Based on literature, Tang et al. [83] measured the expression levels of U6-snRNA, let-7a, miR-21, miR-106a, miR-155, miR-219, miR-221 and miR-16 in the plasma samples of 30 patients with HCC, 30 patients with gastric carcinoma, 20 patients with hepatic cirrhosis, 20 patients with hepatitis B and 20 HC by using qPCR. The expression stability carried out by using specific softwares indicated miR-106a and miR-21 as the best RGs for normalizing this dataset in qPCR assay.

Tay et al. [84] sought to identify endogenous RGs for the study of estrogen-responsive c-miRNAs in human plasma. They analyzed 800 miRNAs in the plasma samples of four healthy males, four non-pregnant females not taking oral contraceptives, four non-pregnant females under oral contraception, and four pregnant females by using nanoString nCounter. From this analysis, 11 miRNAs (miR-25-3p, miR-520f, miR-149-5p, let-7a-5p, miR-2682-5p, miR-612, miR-222-3p, miR-598, miR-188-5p, miR-489 and lastly miR-1183) were selected for a subsequent validation step by qPCR and Digital Droplet PCR (ddPCR) in pooled plasma samples. The results showed that miR-188-5p and miR-222-3p were the best pair of RGs to normalize qPCR data in this experimental set.

Chen et al. [85] investigated the potential diagnostic power of c-miRNAs for CaP. They first performed a microarray analysis to evaluate plasma miRNAs levels in a cohort composed of 25 CaP and 17 benign prostatic hyperplasia (BPH). The identified miRNAs (let-7e, let-7c, miR-346, miR-622, miR-940, miR-1285, miR-25 and miR-30c) were subsequently validated by qPCR in the same population and further confirmed in a greater independent cohort (80 CaP, 44 BPH and 54 HC). To normalize their levels, they examined the stability of miR-16 and U6 which have been reported as RGs in literature. Comparing the Ct values by the Mann-Whitney test, the authors selected U6 as RG to normalize qPCR data, since its expression was more consistent than miR-16. In conclusion, the authors proposed five miRNAs (let-7e, let-7c, miR-622, miR-1285 and miR-30c) as potential biomarkers for CaP, and U6 as the RG to normalize plasma miRNAs levels.

Chen et al. [86] developed a protocol to identify a universal RG. First, a selection phase was carried out by using the Illumina platform to evaluate the serum miRNA levels in 23 pooled serum samples from 130 heterogeneous cancer patients compared to 100 HC. Among the 25 miRNAs selected as candidate RGs according to following criteria: 1. Expressed in all samples, 2. highly expressed, and 3. consistently expressed, the combination of let-7d/g/i resulted in being the most stable. Later, their serum levels and stability, along with other molecules traditionally adopted as RGs according to literature, were evaluated in a dataset of 21 cancer patients by using multiplex qPCR. Based on the results, let-7d/g/i were selected as the best RGs. To increase further data strength, a subsequent validation step was performed by testing the stability of this miRNA combination in a large sample set containing 1278 HC and 777 patients with different types of diseases, and once again their expression levels remained constant. They also showed that these miRNAs were resistant to various harsh conditions (i.e., RNase activity, serum freezing/thawing cycles). Finally, they tested this molecule combination to normalize the expression levels of target spiked-in miRNAs to evaluate their effectiveness for normalizing qPCR data. These results suggested that let-7d/g/i was the best combination for c-miRNA normalization by using qPCR assay. Moreover, they proposed a useful step-by-step experimental procedure for RG identification.

Even the small nuclear RNAs (i.e., RNU1-4, RNU6-2, SNORD43, SNORD44, SNORD48 and SNORA74A) are widely used as RGs for biofluid miRNA expression studies. In this regard, Sanders et al. [87] investigated the previously mentioned RNAs as potential RGs in serum samples of 24 CaP, 12 Non-muscle invasive Bladder Cancer (NMIBC) patients, 12 muscle invasive Bladder Cancer (MIBC) patients, and 24 renal cell carcinoma (RCC) patients by using qPCR. The results showed that SNORD43, alone or combined with RNU1-4, was the most stable RG to study c-miRNAs in these urological malignancies.

## 3. Discussion

One of the major challenges in clinical research is the identification of stable, easily accessible, non-invasive, disease-specific biomarkers that allow physicians to make early diagnosis.

The attention of biofluid miRNAs as biomarkers came after the report of Lawrie et al., which described an up-regulation of miR-21 in the serum of patients with diffuse large B-cell lymphoma in 2008 [23]. Subsequently, a great number of c-miRNAs have been investigated as novel biomarkers for different diseases [21,88,89,90], to such an extent that some research groups are currently evaluating the possible use of miRNA antagonists (antagomirs) as potential therapeutic molecules for specific disease treatment. However, more studies are necessary before they can be used as diagnostic and prognostic tools in clinical practice, and even more so for their use as pharmacological treatment.

In fact, among some technical obstacles that must first be overcome, there is the choice of the most appropriate technologies and protocols with which to perform the analyses, because it is known that each technique for c-miRNAs detection (microarray, NGS, qPCR) affects the outcome of c-miRNA quantification.

Actually, qPCR assay is the most commonly-used approach for miRNAs expression profiling due to its sensitivity, specificity and its low template requirement, and for these reasons it is used for both microarray and NGS data validation [57,58].

However, this method has at least one criticality, regarding accurate RG choice, which is necessary for c-miRNA expression levels to normalize. Indeed, normalization of qPCR data is required for comparative c-miRNA studies across different samples. Initially, the scientific community evaluated the biofluid stability of classic RGs used in intracellular miRNA studies (i.e., U6, 5S, SNORD48, SNORD44, etc.) [91], but they were quickly abandoned due to little stability and susceptibility to degradation. Consequently, xenogeneic or synthetic miRNAs (i.e., ce-miR-39, Quanto EC1, etc.) as exogenous normalization factors, have long been used, adding them to the biofluid sample at a fixed concentration at the beginning of the RNA extraction process.

Recently, the same scientific community has begun to consider these molecules unsuitable as RGs, due to their apparent reduced stability and on the other hand because these exogenous molecules are foreign to biofluid samples, and therefore are not perfectly comparable with endogenous miRNAs. Currently, these molecules are almost exclusively used for the quality and yield control of RNA isolation, cDNA synthesis and PCR amplification [66,84,92].

**Figure 1:**
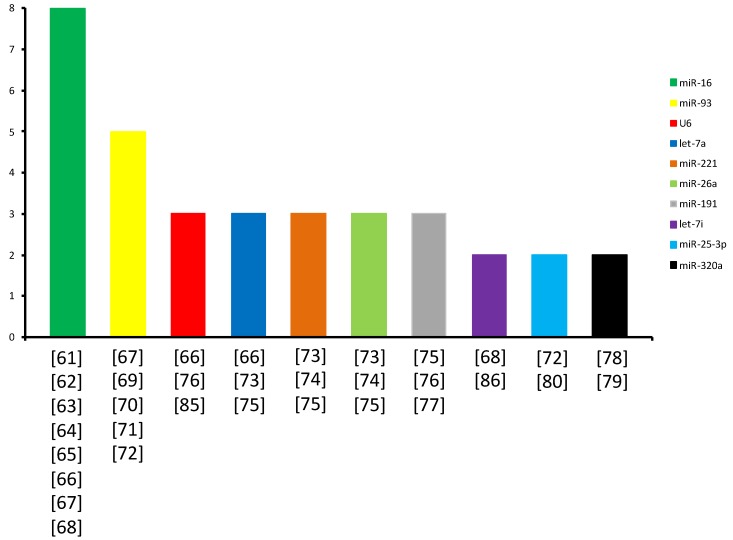
**RGs literature distribution.** The figure shows the RGs ranked according to their usage frequency in literature. miRNAs with frequency = 1 are not shown.

In parallel, numerous research groups are focusing their efforts on identifying specific, highly stable, endogenous c-mRNAs to be used as RGs to normalize biofluid miRNA qPCR-based expression data (Table 1). In Table 2, all the RGs currently used are grouped according to the pathology type for which they were used as RGs. Figure 1 displays their usage frequency, regardless of the pathology studied.

As shown in this review, no consensus exists regarding a single RG constitutively expressed in different types of biofluid samples and pathologies. Endogenous RGs are usually selected either according to previous literature studies or by evaluating the expression of a large miRNA panel in the biofluid samples by using specific platforms. In the first case, after RG selection, the miRNA expression evaluation phase by using qPCR is performed. In the second case, expression data validation is carried out. Finally, a stability assessment by using specific algorithms is performed.

Moreover, some studies have demonstrated that the use of combined RGs increases the efficiency of normalization compared to the use of a single RG.

In conclusion, the choice of suitable RGs is a critical step before starting c-miRNA expression analysis, to guarantee the correct data normalization.

As described so far, there are different approaches to make this choice, but the important thing is that this is supported by solid experimental data that demonstrate the stability robustness of the selected molecules.

This review provides an overview on the use of RGs in the biofluid disease marker field. Accordingly, it could be particularly useful when the choice of RGs to be used in c-miRNAs expression studies is based on literature.

## Figures and Tables

**Table 1:** The best identified non-coding RNAs as potential RGs.

Pathologies	Biological Fluids	Population*	Platforms for miRNA Detection	RG Stability Analysis Methods	RGs	References
Breast Cancer	Plasma	Women with early stage breast (20)	microarray, qPCR		miR-16	[61]
Breast Cancer	Whole blood	Breast cancer (148)	Real-Time qPCR	geNorm	miR-16	[62]
Breast Cancer	Serum	Consecutive breast cancer (83)	qPCR		miR-16	[63]
SMV	Serum	SMV (28)	qPCR	geNorm, NormFinder, Bestkeeper, comparative delta Ct	miR-423-3p, miR-103, miR-16	[64]
Bladder Cancer	Serum	NMIBC (40), MIBC (40), and Bladder cancer (46)	MiSeq sequencing, qPCR	geNorm, NormFinder	miR-193a-5p, miR-16-5p	[65]
Osteogenesis Imperfecta	Serum	osteogenesis imperfecta (22)	qPCR	geNorm, NormFinder, Bestkeeper, comparative delta Ct	snRNAU6/miR-92a, miR-16, let-7a	[66]
Gastric Cancer	Serum	Gastric cancer (20)	qPCR	geNorm, NormFinder, Bestkeeper, comparative delta Ct	miR-16, miR-93	[67]
Cardiovascular Disease	Serum	Cardiovascular disease (35)	qPCR	NormFinder, Bestkeeper, comparative delta Ct	let-7i, miR-16	[68]
Vulvar Carcinoma	Plasma	Vulvar intraepithelial neoplasia lesions (17), and vulvar squamous cell carcinoma (27)	qPCR	geNorm, NormFinder, BestKeeper, comparative delta Ct	miR-93-5p, miR-425-5p	[69]
MDD	Plasma	MDD (32)	Microarray, qPCR	geNorm, NormFinder, BestKeeper, comparative delta Ct	miR-93-5p, miR-101-3p	[70]
Tuberculosis	Plasma	Patients with tuberculosis (24), and patients LTBI (24)	qPCR	geNorm, NormFinder	miR-93	[71]
Colorectal Cancer	Serum	Colorectal cancer (96)	Microarray, qPCR	geNorm, NormFinder, BestKeeper	miR-93-5p, miR-25-3p, miR-106b-5p	[72]
Liver Carcinoma	Serum Exosomal	Pre-operative and post-operative patients with HCC (53)	qPCR	geNorm, NormFinder, BestKeeper, comparative delta Ct	miR-221, miR-26a, let-7a	[73]
HBV	Serum	HBV (52)	TaqMan low density arrays, qPCR	geNorm, NormFinder	miR-26a, miR-221, miR-22*	[74]
HBV and HCC	Serum Exosomal	HCC (50), and HBV (50)	Real-Time qPCR	geNorm, NormFinder	miR-221, miR-191, let-7a, miR-181a, miR-26a	[75]
Colorectal Adenocarcinoma	Serum	Colorectal adenocarcinoma (203)	Miseq sequencing, qPCR	geNorm, NormFinder	miR-191-5p, U6	[76]
Breast Cancer	Serum	Breast cancer (225), lung cancer (65), cervical cancer (30), gastric cancer (45), HCC (35), esophageal cancer (5), colon cancer (5), rectal cancer (5), pancreatic cancer (5), and oral cancer (5)	Solexa sequencing, TaqMan low density array, qPCR	NormFinder	miR-191, miR-484	[77]
Osteosarcoma	Plasma	Engineered mouse models of osteosarcoma, osteosarcoma human patients (60)	qPCR	geNorm	miR-320a, miR-15a	[78]
PAH or Septic Shock	Plasma	PAH (18), and patients with septic shock (4)	qPCR	NormFinder	miR-142-3p, miR-320a	[79]
Osteoporosis	Serum	Osteoporosis-induced animal model; normal BMD (19), osteopenic (7), and osteoporosis woman (10)	Microarray, qPCR	geNorm, NormFinder	miR-25-3p	[80]
Different Cancer Types	Plasma	HCC (88), colorectal cancer (62), lung cancer (103), esophageal cancer (23), gastric cancer (21), renal cancer (24), CaP (20), and breast cancer (21)	Microarray, qPCR	geNorm, NormFinder	miR-1228	[81]
CAD	Plasma	CAD (119)	Microarray, qPCR	NormFinder, BestKeeper	miR-6090, miR-4516	[82]
HCC	Plasma	HCC (30), gastric carcinoma (30), hepatic cirrhosis (20), and HBV (20)	qPCR	geNorm, NormFinder, BestKeeper, comparative delta Ct	miR-106a, miR-21	[83]
Protein S Deficiency in Pregnancy	Plasma	Non-pregnant females not taking oral contraceptives (14), non-pregnant females currently taking oral contraceptives (14), and pregnant females (14)	nanoString nCounter, qPCR, Digital Droplet PCR	NormFinder, Bestkeeper	miR-188-5p, miR-222-3p	[84]
CaP	Plasma	CaP (105), and benign prostatic hyperplasia (61)	qPCR	comparative delta Ct	U6	[85]
Different Pathology Types	Serum	Non-Small Cell Lung Cancer (87), pancreatic cancer (88), gastric cancer (87), esophageal cancer (46), colorectal cancer (30), patients with HCC (30), breast cancer (58), ovarian cancer (26), cervical cancer (40), nephritis (101), colitis (73), pancreatitis (18), pneumonia (8), and type 2 diabetes (320)	Illumina technology, qPCR	geNorm, NormFinder	let-7d/g/i	[86]
CaP, Bladder Cancer and RCC	Serum	CaP (24), NMIBC (12), MIBC (12), and RCC (24)	qPCR	geNorm, NormFinder, comparative delta Ct	SNORD43, RNU1-4	[87]

* In brackets, the number of recruited patients.

**Table 2:** RGs Pathology-based RG groups.

Pathology	RGs	References
Breast Cancer	miR-16	[61]
	miR-16	[62]
	miR-16	[63]
	miR-191, miR-484	[77]
	miR-1228	[81]
	let-7d/g/i	[86]
Urogenital Malignances (Bladder, Prostate, Renal)	miR-16-5p, miR-193a-5p	[65]
	miR-1228	[81]
	U6	[85]
	SNORD43, RNU1-4	[87]
Bone Diseases (Osteogenesis imperfecta, Osteosarcoma, Osteoporosis)	miR-16, U6, miR-92a, let-7a	[66]
	miR-320a, miR-15a	[78]
	miR-25-3p	[80]
Gastrointestinal Disorders (Esophagus Cancer, Colorectal Cancer, Gastric, Cancer, Pancreatic Disease)	miR-93, miR-16	[67]
	miR-93-5p, miR-25-3p, miR-106b-5p	[72]
	miR-191-5p, U6	[76]
	miR-1228	[81]
	let-7d/g/i	[86]
Cardiovascular Pathologies(Hypertension, Heart Failure, CAD)	miR-16, let-7i	[68]
	miR-6090, miR-4516	[82]
Female Reproductive System Cancers(Vulvar, Ovarian, Cervical)	miR-93-5p, miR-425-5p	[69]
	let-7d/g/i	[86]
Lung Diseases(Tuberculosis, Pulmonary cancers)	miR-93	[71]
	miR-320a, miR-142-3p	[79]
	miR-1228	[81]
Liver Disorders	miR-221, let-7a, miR-26a	[73]
	miR-221, miR-22*, miR-26a	[74]
	miR-221, let-7a, miR-191, miR-26a, miR-181a	[75]
	miR-1228	[81]
	miR-106a, miR-21	[83]
	let-7d/g/i	[86]
MDD	miR-93-5p, miR-101-3p	[70]
SMV	miR-423-3p, miR-103, miR-16	[64]
Protein S deficiency in pregnancy	miR-188-5p and miR-222-3p	[84]
